# Integrating an NTD with One of “The Big Three”: Combined Malaria and Trachoma Survey in Amhara Region of Ethiopia

**DOI:** 10.1371/journal.pntd.0000197

**Published:** 2008-03-19

**Authors:** Paul M. Emerson, Jeremiah Ngondi, Estifanos Biru, Patricia M. Graves, Yeshewamebrat Ejigsemahu, Teshome Gebre, Tekola Endeshaw, Asrat Genet, Aryc W. Mosher, Mulat Zerihun, Ayennew Messele, Frank O. Richards

**Affiliations:** 1 The Carter Center, Atlanta, Georgia, United States of America; 2 Department of Public Health and Primary Care, University of Cambridge, Cambridge, United Kingdom; 3 The Carter Center, Addis Ababa, Ethiopia; 4 Amhara Regional Health Bureau, Bahir Dar, Ethiopia; 5 The Carter Center, Bahir Dar, Amhara National Regional State, Ethiopia; University of California San Francisco, United States of America

## Abstract

**Background:**

Amhara Regional State of Ethiopia has a population of approximately 19.6 million, is prone to unstable and epidemic malaria, and is severely affected by trachoma. An integrated malaria and trachoma control program is being implemented by the Regional Health Bureau. To provide baseline data, a survey was conducted during December 2006 to estimate malaria parasite prevalence, malaria indicators, prevalence of trachoma, and trachoma risk factors in households and people of all ages in each of the ten zones of the state, excluding three urban centers (0.4% of the population).

**Methodology/Principal Findings:**

The study was designed to provide prevalence estimates at zone and state levels. Using multi-stage cluster random sampling, 16 clusters of 25 households were randomly selected in each of the ten zones. Household heads were interviewed for malaria indicators and trachoma risk factors (N = 4,101). All people were examined for trachoma signs (N = 17,242), and those in even-numbered households provided blood films for malaria parasite detection (N = 7,745); both thick and thin blood films were read.

Zonal malaria parasite prevalence ranged from 2.4% to 6.1%, with the overall state-wide prevalence being 4.6% (95% confidence interval (CI): 3.8%–5.6%). The *Plasmodium falciparum*: *Plasmodium vivax* ratio ranged from 0.9–2.1 with an overall regional ratio of 1.2. A total of 14.8% of households reported indoor residual spraying in the past year, 34.7% had at least one mosquito net, and 16.1% had one or more long-lasting insecticidal net. Zonal trachoma prevalence (trachomatous inflammation follicular [WHO grade TF] in children aged 1–9 years) ranged from 12.6% to 60.1%, with the overall state-wide prevalence being 32.7% (95% CI: 29.2%–36.5%). State-wide prevalence of trachomatous trichiasis (TT) in persons aged over fifteen was 6.2% (95% CI: 5.3–7.4), and 0.3% (95% CI: 0.2–0.5) in children aged 0–14 years. Overall, an estimated 643,904 persons (lower bound 419,274, upper bound 975,635) have TT and require immediate corrective surgery.

**Conclusions/Significance:**

The results provide extensive baseline data to guide planning, implementation, and evaluation of the integrated malaria and trachoma control program in Amhara. The success of the integrated survey is the first step towards demonstration that control of priority neglected tropical diseases can be integrated with one of the “big three” killer diseases.

## Author Summary

The “big three” killer diseases are malaria, HIV/AIDS, and tuberculosis; control programs for these diseases are usually well developed and financed. The neglected tropical diseases (NTDs) are a group of ancient afflictions that are frequently sidelined by planners and are under-resourced. Opportunities of integrating the big three with NTDs have been talked about but not widely acted upon. There is potential synergy for an integrated trachoma and malaria control program since control of both diseases is community-based. The first step in accessing these synergies has been an integrated malaria prevalence and indicator and trachoma prevalence and risk factor survey. This has been achieved at the incremental cost of one additional staff member per field team. The results give unprecedented precision for the calculation of intervention targets for the integrated program and demonstrate that it is possible to integrate NTDs with the “big three.”

## Introduction

Ethiopia is a rapidly developing country that is burdened and held back by a high prevalence of communicable disease. Of the so-called ‘big three’ killer diseases, HIV/AIDs, tuberculosis, and malaria, malaria is the most frequent cause of out-patient presentation and in-patient admission nationwide and is second only to respiratory tract infections as a cause of death in children [1]. Co-endemic with the ‘big three’ in Ethiopia are the ‘neglected tropical diseases’ of which trachoma is the most geographically widespread and cause of greatest morbidity.

The Ethiopian national blindness and low vision survey conducted in 2006 suggests that Ethiopia is the most trachoma affected country in the world. The entire rural population of approximately 65 million people are at risk of blindness from trachoma. At any time there are an estimated 9 million children with clinical signs of active disease, 1.2 million adults with trachomatous trichiasis, and 354,000 persons with blindness or low vision attributed to trachoma [2]. In addition to the effects on vision and the high likelihood of developing blindness if unoperated [3], trichiasis is a terrible condition in which the eyelashes rub against the surface of the eye ball, leaving sufferers in constant and disabling pain. The irritation and pain caused by the lashes on the surface of the eye ball and cornea is exacerbated by smoke, dust and bright light which prevents people from conducting their normal routine activities such as cooking over solid fuel fires, farming in dusty environments, gathering firewood and collecting water [4]. Of the ten states in Ethiopia, Amhara Regional State is disproportionately affected by trachoma, bearing an estimated minimum of 45% of the national trichiasis burden and with approximately one in twenty of all adults suffering from trichiasis [2]. It is recognized that the geographic distribution of trachoma within the state is not uniform with some health posts reporting trachoma as the primary cause of out-patient consultations and for others, trachoma ranks below malaria and respiratory tract infection as a cause of out-patient consultation. Trachoma is a barrier to development in Amhara and controlling this disease is a state priority.

Trachoma control in Amhara, and the whole of Ethiopia, is based on the World Health Organization (WHO) endorsed SAFE strategy, in which S is corrective lid surgery for patients with trichiasis, A is antibiotic treatment for individuals with signs of active disease and for mass drug administration to at-risk populations, F is facial cleanliness and hygiene promotion to prevent transmission, and E is environmental improvements such as provision of sanitation and water that address trachoma risk factors [5].

Nationally, it has been recognized that malaria is a major barrier to the development. Ethiopia including Amhara , is prone to unstable and epidemic malaria [6],[7],[8]. The Federal Ministry of Health has launched a control program of unprecedented scale and scope to relieve this burden. The program is based on personal protection/vector control, and effective case detection and treatment. It is being targeted to the entire Ethiopian population at risk of malaria (estimated at 50 million people) [9]. The program has four pillars: distribution of free long-lasting insecticidal nets (LLINs) at the target rate of two per household in malaria-endemic areas; targeted indoor residual spraying (IRS) in high transmission areas; serum-based rapid diagnostic tests (RDTs) available at all health facilities; and treatment with artemesinin combination therapy for *Plasmodium falciparum* malaria and chloroquine for uncomplicated malaria caused by *Plasmodium vivax*. In Amhara, the malaria is seasonal and follows the rain; presentation of malaria cases is most common from September to January, with a peak in November and December. The survey was conducted in December at the end of the normal peak transmission period.

Recently, there have been a series of publications proposing that large single disease programs be integrated to improve efficiency and reduce costs [10], that programs specifically targeting the neglected tropical diseases be integrated and expanded [11], that neglected tropical disease programs be integrated with the ‘big three’ [12], and that deworming programs be integrated with malaria control [13]. It was in this climate that the Amhara Regional Health Bureau partnered with The Carter Center to plan an integrated malaria and trachoma control program that would simultaneously target two of the most devastating communicable diseases in the state: malaria and trachoma.

**Figure 1:**
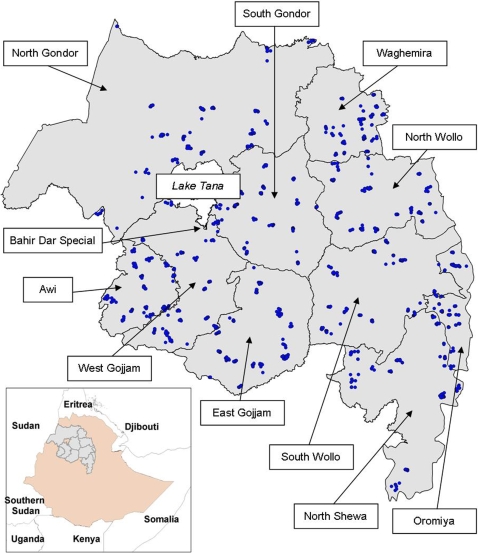
Map of Amhara Region of Ethiopia showing the survey sites.

Amhara (population approximately 19.6 million [14]) is divided administratively into 10 zones, which are themselves divided into 140 woredas (district equivalents), and 3,231 kebeles (groups of villages with approximately 5,000 population) (Figure 1, Map). Within kebeles the lowest administrative unit is the state team (now known as development teams) which are groups of about 50 families, usually around 250 people, who have an elected representative. Although national direction comes from the Federal Ministry of Health, overall coordination of programs has been devolved to the states, and planning for implementation is conducted by the woreda representatives at the level of the zone. In order to facilitate planning of the interventions and to enable evaluation at both state and zonal level, we conducted an integrated malaria and trachoma survey that was powered to provide prevalence estimates at the zonal level. The integrated survey had the following objectives.

*Malaria:* 1) Estimate malaria parasite prevalence by species of parasite and age and gender of host at the zonal level; and 2) Estimate the coverage of indoor residual spraying, use of mosquito nets and use of long-lasting insecticidal nets.

*Trachoma:* 3) Estimate the prevalence of trachomatous inflammation follicular (WHO grade TF) in children aged 1–9 years at the zonal level; 4) Estimate prevalence of trachomatous trichiasis (WHO grade TT) in adults aged over 15 years by zone and sex; and 5) Estimate other household risk factors for trachoma including latrine ownership, access to water supply, and facial cleanliness of children aged 1–9 years.

The data from the integrated survey would allow calculation of programmatic needs for both components of the integrated program: malaria and trachoma. For malaria the number of long lasting insecticidal nets required to meet the distribution targets and information on current net usage for targeting health education messages; for trachoma the estimated backlog of TT surgeries, the population requiring mass drug administration with antibiotic and health education, and the need for household latrines and access to water.

## Methods

### Sample size calculation

The sample size was calculated based on the assumption that prevalence of TT would be the lowest of the indicators that we wished to measure and we would determine prevalence estimates at the zonal level. For each zone we assumed a prevalence of TT in adults of 5%, 2.0% precision, 95% confidence limit, and a design effect of 2. We estimated that we needed to examine at lest 1,000 adults per zone. We assumed adults were 50% of the population, so that the total population sampled would need to be 2,000, and that household size was, on average, 5. This gives an estimated sample of 400 households per zone.

Bahir Dar town was excluded from the sampling frame as were two special woredas ‘Debre Markos Ketema’ in East Gojjam and ‘Debre Tabor’ in South Gondar zone. The latter two were excluded because, in accordance with the Regional Health Bureau definition, less than 10% of their population lived in malarious areas; however, these two woredas comprised only 0.4% of the overall population.

To select the 400 households in each zone we used a multi-stage cluster random sampling design. In each zone, eight woredas were selected using probability proportional to size and, within the selected woredas, we selected two kebeles also using probability proportional to size. Within the kebele, five state teams (which are all approximately similar in size) were selected by lottery, literally drawing the names out of a hat at the woreda office. In the final stage, five households were selected from the 50 in the state team using the random walk method.

To determine malaria parasite prevalence, we assumed a population prevalence of 8%, 2% precision, 5% level of significance and 95% confidence limit, a design effect of 1.2 and 15% non-response to give an estimated sample size of 1,000 people or 200 households in each zone. Consequently, we arbitrarily determined that even-numbered households in the overall sample would be recruited for malaria blood films.

In summary, the integrated survey had ten domains (the zones). Within each zone there were 16 clusters, each of 25 households to give a sample of 400 households or 2,000 people per zone. Overall there were 160 clusters of 25 households. Each zone had a different population, so that persons sampled within a zone represented a different proportion of the total population. State-wide prevalence estimates could be calculated from the weighted domain estimates. The survey was conducted in December 2006 at the end of the malaria transmission season.

### Household questionnaire

The survey questionnaire was based on the Malaria Indicator Survey Household Questionnaire , modified for the local conditions and to include risk factors for trachoma [15]. The questionnaire was translated and printed in Amharic language and field-tested in a non-survey kebele to determine the validity of the pre-coded answers. Interviews were conducted with the head of household, or another adult if the head of household was absent or unable to respond for any reason. If interviews were conducted with someone other than the head of household then the respondent was requested to answer as though he or she were the head of household. The data collection form had three parts: household questionnaire; malaria parasite prevalence; and trachoma survey.

In the household questionnaire respondents were asked about: their source of drinking water; time to collect water; toilet facilities (latrine presence, if reported, was verified by observation); proxy indicators of wealth; room construction materials; indoor residual spraying; presence and type of mosquito net (verified by observation); demographic information on residents; and where people slept. We defined the source of drinking water as being ‘safe’ if it was a capped spring, protected hand-dug well, tube well, borehole, cart with small tank, or piped water. Other water sources were described as ‘unsafe’ and were unprotected springs, unprotected hand-dug wells, and surface water. Our proxy indicators of wealth were electrification of the household, possession of a functioning radio set, and possession of a functioning television set.

### Malaria parasite prevalence

Consenting residents of even-numbered households were recruited for the malaria parasite prevalence survey. Participants had both a rapid diagnostic test which gave an on-the-spot diagnosis and provided thick and thin blood films for microscopy. The rapid diagnostic test used was ParaScreen (Zephyr Biomedical Systems, www.tulipgroup.com), this test is able to detect both *P. falciparum* and other plasmodia species (in Amhara most likely *P. vivax*). The test uses approximately 100 µl of blood and is readable after ten minutes. Participants with positive rapid tests were offered treatment according to national guidelines, CoArtem® for *P. falciparum* infection, chloroquine for other *Plasmodium* infection, and clinic-based quinine therapy for self-reported pregnant women [16].

Two blood slides, each composed of thick and thin films, were taken for each participant by a clinical technician according to standard WHO-approved protocol [17]. Slides were labelled and air-dried horizontally in a carrying case in the field, and stained with Giemsa at the nearest health facility when the team returned from the field. Usually, field teams returned to the clinic each evening but when working in inaccessible areas, which required walking up to eight hours each way, they were obliged to sleep in the field and stain the slides the following day. To ensure maximum participation, households with absentees were revisited a second time on the same day to recruit those missing at the first visit.

Blood slides were read at a reference laboratory in Addis Ababa and classified qualitatively as either negative, *P. falciparum* positive, *P.vivax* positive, or mixed infection. One hundred high power fields of the thick film were examined before calling a slide negative. If positive, the thin film was read to determine the species. Parasite density was not quantified. To ensure accuracy, all positive slides and a random sample of 5% of the negative slides were re-examined by a separate microscopist, who was blinded to the diagnosis of the first slide-reader. The second slide from each participant was used if the first was broken or unreadable. The identity of survey participants who had positive blood slides was sent back to the field teams for follow-up and appropriate treatment, where necessary.

### Trachoma survey

Trachoma grading was carried out by Integrated Eye Care Workers (IECW) who were experienced in using the WHO simplified grading [18]. This scheme comprises of 5 stages: trachomatous inflammation-follicular (TF), trachomatous inflammation-intense (TI), trachomatous scarring (TS), trachomatous trichiasis (TT) and corneal opacity (CO) (Box 1). Minimum accepted inter-observer agreement was set at 80% and reliability assessed in two stages. In the first stage, potential examiners identified trachoma grades using the WHO set of trachoma slides [19]. Those examiners who achieved at least 80% agreement then proceeded to the second stage of field evaluation. During field evaluation a reliability study comprising 50 persons of varying age and sex were selected by the senior examiner to represent all trachoma grades. Each potential examiner evaluated all 50 subjects independently and recorded their findings on a pre-printed form. Inter-observer agreement was then calculated for each trainee using the senior examiner's observation as the ‘gold standard’. Examiners achieving at least 80% inter-observer agreement after the field evaluation were included as graders.

All persons living within each selected household who gave verbal consent were examined using a torch and a x2.5 magnifying binocular loupe. Each eye was examined first for in-turned lashes (TT), and the cornea was then inspected for corneal opacities (CO). The upper conjunctiva was subsequently everted and examined for inflammation (TF and TI) and scarring (TS). Both eyes were examined. Signs had to be clearly visible in accordance with the simplified grading system in order to be considered present. Alcohol-soaked cotton-swabs were used to clean the examiner's fingers between examinations. Individuals with signs of active trachoma (TF and/or TI) were offered treatment with 1% tetracycline eye ointment. TT patients were referred to health centres where free eyelid surgery was available.

Data to determine whether children aged 1–9 years had a ‘clean face’ were collected during the eye examinations. Facing the child, the observer looked for the presence of ocular and nasal discharge, recording each separately as a dichotomous variable. Ocular discharge was defined as any material of any colour or consistency in the corner of the eyes, or matting of the eyelashes caused by such a discharge (tears, medication and make-up were excluded). Nasal discharge was defined as the presence of wet exudate of any colour below one or both nostrils.

### Quality control, data entry and analysis

Forms were checked by the supervisor in the field and inconsistencies verified with the respondent. Data were double entered by different entry clerks and compared for consistency using Census and Survey Processing System (U.S. Census Bureau Washington DC, USA). Statistical analysis was conducted using Stata™ 9.2 (Stata Corporation, College Station, Texas, USA). Descriptive statistics were used to describe the characteristics of the sample. Sampling probabilities were calculated for woredas, kebeles and state teams. Sampling weights were then derived as the inverse of the product of sampling probabilities at the woreda, kebele and state team levels. Point estimates and confidence intervals were derived using the SURVEY (SVY) routine in Stata which controls for clustering and allowed for adjustments for the sampling design as well as weighting for sampling probability [20].

To give greater precision in the estimates of trichiasis burden, TT prevalence was modelled for sex-specific ten-year age groups using logistic regression. Prevalence of TT was calculated for ten-year age groups for males and females separately for each zone. Ten-year age population structures by sex were obtained for each zone from the Amhara Regional Health Bureau [14] and applied to the ten-year age group TT prevalence estimates for males and females. The 95% confidence intervals of the point prevalence estimates were multiplied by the respective population structure estimates to derive the lower and upper bounds of the TT burden. All zonal estimates and corresponding upper and lower bounds were summed to derive the state-wide estimate of those requiring TT surgery.

### Ethical consideration

The protocol received ethical approval from the Emory University Institutional Review Board (IRB 1816) and the Amhara Regional Health Bureau. Verbal informed consent to participate in interviews and trachoma screening was sought from the heads of the household, each individual and the parents of children aged 10 years and younger in accordance with the tenets of the declaration of Helsinki. Signed informed consent was sought from each individual and parents of children aged 10 years and younger in accordance with the tenets of the declaration of Helsinki for blood films. Personal identifiers were removed from the data set before analyses were undertaken.

## Results

### Characteristics of study households and participants

**Figure 2:**
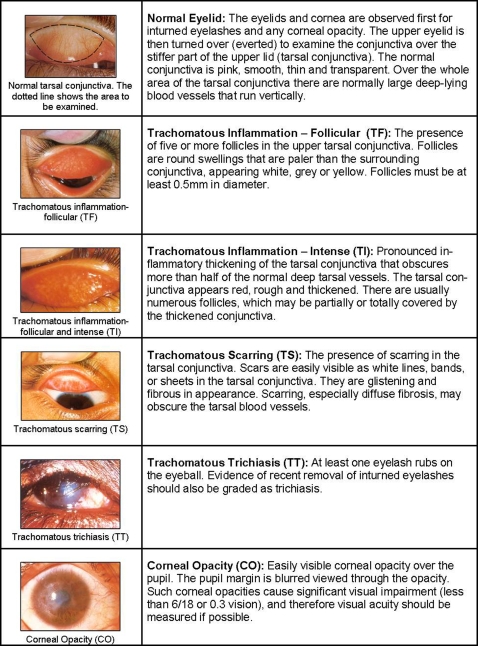
The WHO simplified grading scheme for assessment of trachoma. Reproduced from Thylefors, 1987 [18].

A total of 4,122 households were selected for the survey of which 21 were excluded since no one was present: 4,101 (99.5%) were included in the analysis (Figure 2). The characteristics of the study households are shown in Table 1, and the location of the sites surveyed are represented on Figure 1. The overall mean household size was 4.6 persons (95% confidence interval [CI] 4.5–4.7) with household size ranging from 1 to 17. The main source of drinking water came from ‘safe’ sources for 34.4% (95% CI 27.8–64.3) of households and the round-trip time to collect water was <30 minutes for 74.1% (95% CI 69.1–78.5). Household pit latrines were present in 24.3% (95% CI 19.9–29.3) of households with 75.7% (95% CI 70.7–80.1) reporting open defecation. Our proxy indicators of wealth (electricity, radio, TV) were reported in 5.7% (95% CI 3.0–10.4), 24.8% (95% CI 22.2–27.6), 1.2% (95% CI 0.8–2.0) of households, respectively. The majority of households, 57.1% (95% CI 52.3–61.8), had a thatch roof; walls made from sticks, 90.5% (95% CI 87.2–93.0); and floors of compacted earth, 73.3% (95% CI 68.8–77.4). Indoor residual spraying in the last 12 months was reported in 14.8% (95% CI 10.1–21.1) of households. Any mosquito net was reported and verified by observation in 34.7% (95% CI 28.3–41.6) of households with a range of 0 to 5 nets observed. Of all the nets seen in all the households, 54.6% were LLINs, and 16.1% (95% CI 12.1–21.2) of households had at least one LLIN. The mean number of LLINs per household was 0.30 (95% CI 0.2–0.3).[Fig pntd-0000197-g003]

**Figure 3 pntd-0000197-g003:**
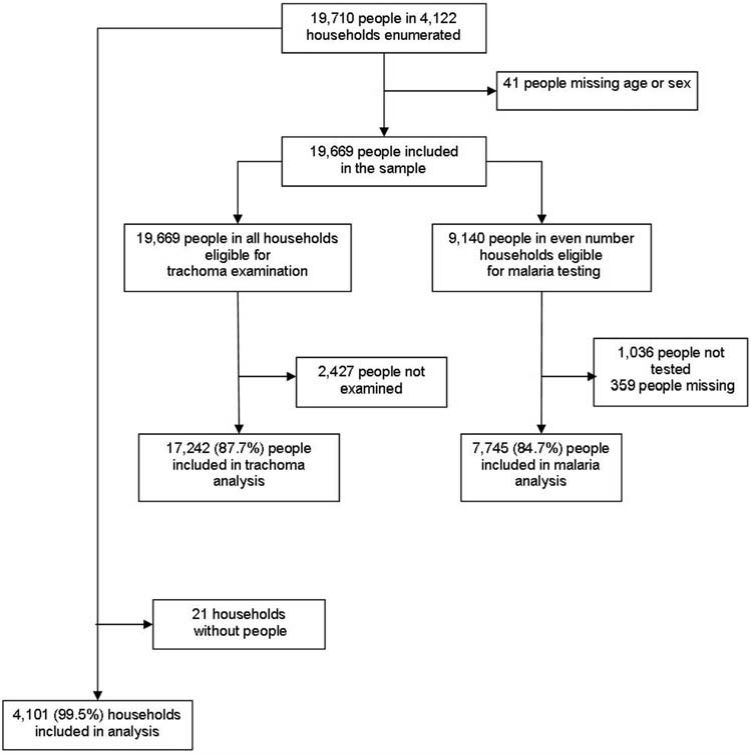
The sample population.

**Table 1:** Characteristics of study households

			Household characteristics								
Domain	Estimated population*	Clusters sampled	Number sampled	Size Mean	Thatch roof (%)	Without pit latrine (%)	Water source > = 30 minutes (%)	Mosquito nets			Insecticide sprayed in the last year (%)
								Any net %	LLIN %	Mean number of LLIN per HH	
Amhara Region	19,391,698	160	4,101	4.6	57.1	75.7	25.9	34.7	16.1	0.3	14.8
Zones											
North Gondor	3,241,161	16	392	4.7	55.1	85.4	60.3	50.5	33.4	0.5	9.8
Waghemira	375,440	16	410	5.0	91.7	94.3	53.1	52.8	52.0	0.8	8.4
South Gondor	2,243,477	16	415	5.0	74.0	76.6	3.6	55.6	4.1	0.1	13.6
North Wollo	1,636,699	16	404	4.5	79.1	49.8	12.8	47.2	30.1	0.4	16.6
West Gojjam	2,674,974	16	400	4.5	15.8	82.8	39.0	33.8	13.7	0.2	24.5
Awi	1,090,879	16	395	5.0	46.7	54.1	25.7	21.3	9.4	0.1	13.7
East Gojjam	2,470,060	16	459	4.2	21.7	81.6	26.7	5.6	0	0	8.8
South Wollo	2,878,970	16	403	4.1	81.5	74.2	10.8	6.1	4.0	0.04	2.5
Oromiya	588,943	16	400	5.8	76.9	59.4	36.1	99.9	53.6	1.2	57.4
North Shewa	2,191,096	16	423	5.2	62.4	91.1	21.4	19.2	11.2	0.2	9.3

*: Bureau of Finance and Economic Development for the year 2006/2007

HH, household; LLIN, Long lasting insecticidal net

Table 2 shows the individual characteristics of study participants and mosquito net usage. A total of 19,710 people were enumerated of whom 41 were excluded from analysis due to missing data on age or sex. Of the 19,669 people included in the sample, the overall mean age was 21.9 years (95% CI 21.5–22.3) and 48.7% were male. The overall proportion of people reporting sleeping under any mosquito net the previous night was 25.8% (95% CI 21.2–30.9). Among the population with particular vulnerability to malaria, the under five year-olds and pregnant women, sleeping under a mosquito net was reported for 29.2% (95% CI 24.1–34.9) and 33.6% (95% CI 25.4–42.8), respectively. Persons reporting sleeping under an LLIN the previous night were: 12.5% (95% CI 9.4–16.5) overall. Of the vulnerable groups, 425/2,929 children aged less than 5 years, 14.5% (95% CI 10.8–19.2); and 46/315 pregnant women, 14.6% (95% CI 10.0–21.0) reported sleeping under an LLIN last night.

**Table 2:** Characteristics of study participants and net usage

	Participants characteristics				Proportion of people reporting sleeping under a net last night (%)					
Domain	Number included in the sample	Mean Age	Male (%)	Unclean face in children (%)**	Slept under any net			Slept under LLIN		
					All people	Under fives	Pregnant women	All people	Under fives	Pregnant women
Amhara Region	19,669	21.9	48.7	25.9	25.8	29.2	33.6	12.5	14.5	14.6
Zones										
North Gondor	1,892	20.5	47.8	18.6	34.3	38.7	62.4	23.0	27.4	38.1
Waghemira	2,018	21.7	51.2	53.6	38.7	45.5	78.8	37.6	43.9	77.3
South Gondor	2,094	21.0	52.3	24.1	37.3	39.0	59.5	1.7	2.4	2.0
North Wollo	1,812	23.3	48.5	32.9	31.0	31.8	0.0	21.6	20.6	0.0
West Gojjam	1,812	21.2	47.7	25.3	21.3	24.3	27.3	8.5	10.4	18.5
Awi	1,965	20.2	48.6	74.9	15.5	18.1	10.6	7.4	7.2	4.1
East Gojjam	1,949	22.2	45.6	42.8	2.1	4.0	6.4	0.0	0.0	0.0
South Wollo	1,653	23.6	48.1	3.2	4.5	4.1	14.7	2.9	2.1	4.8
Oromiya	2,289	20.7	48.9	22.2	91.7	97.1	96.5	48.3	51.1	43.6
North Shewa	2,185	23.0	48.9	23.7	14.3	14.8	12.6	8.7	8.6	3.5

LLIN, Long lasting insecticidal net

Figure 2 shows the sample population and those recruited for trachoma examination and malaria parasite prevalence. Trachoma examination was conducted in 17,242 (87.7%). A total of 9,140 people in even numbered households were eligible for malaria testing of whom 7,745 were included in the analysis (84.7%).

### Malaria prevalence

The malaria parasite prevalence by blood slide microscopy is shown on Table 3. A total of 7,745 blood slides were examined with good concordance between first and second reading. The overall malaria parasite prevalence in Amhara was 4.6% (95% CI 3.8–5.6) with prevalence by zone ranging from 2.4% (95% CI 1.5–4.0) in Oromiya to 6.1% (95% CI 4.5–8.5) in South Gondor. There were no differences in the proportion of people with positive blood slides by age group: age <5 years, 5.4%; age 5–14 years, 3.8%; age 15–49 years, 4.5%; and age >50 years, 4.2%. The malaria species seen most frequently was *P. falciparum*, 52.2% of positive slides had *P. falciparum* only and 8.7% were mixed *P. falciparum and P. vivax*. *Plasmodium vivax* only was seen on 41.3% of the positive slides. The overall ratio of *P. falciparum* to *P. vivax* was 1.2 with zonal estimates ranging from 0.9 to 2.1.

**Table 3:** Prevalence of malaria by blood slide microscopy

Domain	Number examined	Malaria parasite prevalence (%)				Pf:Pv ratio
		*P.falciparum* only	*P.vivax* only	Mixed Pf and Pv	Total* % (95% CI)	
Amhara Region	7,745	2.4	1.9	0.4	4.6 (3.8–5.6)	1.2
Zones						
North Gondor	703	3.1	2.8	0.0	5.9 (4.2–8.2)	1.1
Waghemira	809	2.0	1.0	0.2	3.1 (1.5–6.4)	1.9
South Gondor	744	2.7	2.5	0.8	6.1 (4.3–8.5)	1.1
North Wollo	835	1.3	1.5	0.4	3.1 (1.5–6.2)	0.9
West Gojjam	713	1.5	1.7	0.3	3.4 (1.7–6.7)	0.9
Awi	799	2.4	2.8	0.4	5.6 (2.4–12.3)	0.9
East Gojjam	699	2.9	1.6	0.4	4.9 (2.4–9.6)	1.7
South Wollo	758	3.6	1.5	0.5	5.6 (3.2–9.4)	2.1
Oromiya	825	1.0	1.1	0.3	2.4 (1.5–4.0)	0.9
North Shewa	860	2.6	2.0	0.2	4.8 (2.7–8.4)	1.3

CI, confidence interval; Pf, *Plasmodium falciparum*; Pv, *Plasmodium vivax*

*: Total parasite prevalence do not equal the sum of the species prevalence in some rows due to rounding

### Trachoma prevalence

The key trachoma prevalence indicators are shown in Table 4. The overall prevalence of TF in children aged 1–9 years was 32.7% (95% CI 29.2–36.5). Prevalence of TF in children by zone ranged from 12.6% (95% CI 7.8–19.7) in South Wollo to 60.1% (95% CI 50.4–69.0) in Waghemira. There was no sex difference in TF prevalence: Odds Ratio (OR) = 1.0 (95% CI 0.9–1.2). The overall prevalence of TT in persons aged 15 years and above was 6.2% (95% CI 5.3–7.4). Point estimates by zone of TT prevalence in adults ranged from 2.4% (95% CI 1.4–4.1) in Oromiya to 10.0% (95% CI 6.3–15.6)in West Gojjam. After adjusting for age, adult women were three times more likely to have TT than men: OR = 3.1; (95% CI 2.3–4.1). Trichiasis was also observed in children aged less than 15 years with an overall prevalence of 0.3% (95% CI 0.2–0.5) in the age group 0–14 years, ranging from 0% in North Gondor to 0.8% (95% CI 0.3–1.8) in North Wollo.

**Table 4:** Key trachoma prevalence indicators: trachomatous inflammation–follicular (TF) and trachomatous trichiasis (TT)

Domain	TF in children aged 1–9 years			TT in children aged 0–14 years			TT in people aged 15 and above		
	Number examined	Prevalence		Number examined	Prevalence		Number examined	Prevalence	
		%	95% CI		%	95% CI		%	95% CI
Amhara Region	5,485	32.7	29.2–36.5	8,121	0.3	0.2–0.5	9,121	6.2	5.3–7.4
Zones									
North Gondor	466	34.7	24.4–46.8	700	0		730	4.3	2.8–6.6
Waghemira	581	60.1	50.4–69.0	918	0.5	0.2–1.5	1,030	6.3	3.9–9.9
South Gondor	589	28.9	20.1–39.6	887	0.1	0.01–0.4	904	3.8	2.5–5.7
North Wollo	539	51.9	35.4–68.0	739	0.8	0.3–1.8	971	9.4	7.2–12.1
West Gojjam	500	33.1	25.3–42.0	774	0.4	0.1–1.3	874	10.0	6.3–15.6
Awi	588	38.9	22.7–57.9	866	0.1	0.01–0.4	893	5.4	4.0–7.3
East Gojjam	548	48.3	44.4–52.2	798	0.3	0.1–0.8	881	7.1	5.4–9.4
South Wollo	484	12.6	7.8–19.7	701	0.3	0.1–1.4	931	3.2	2.2–4.6
Oromiya	663	28.7	19.6–39.8	958	0.1	0.02–0.8	964	2.4	1.4–4.1
North Shewa	527	23.2	14.1–35.9	780	0.3	0.1–1.1	943	9.0	6.7–11.9

CI, confidence interval

### Trichiasis burden estimates

Estimates of trichiasis burden are summarized in Table 5. The number of people with TT in Amhara was estimated to be 643,904 (lower and upper bounds = 419,274–975,635). Consistent with the increased odds of TT in women, the TT burden in females (of all ages) was estimated to be 2.2 fold compared to males. For planning purposes, the number of persons requiring corrective trichiasis surgery in Amhara was estimated to be 645,000.

**Table 5:** Trichiasis burden estimates by gender

Domain	Male			Female			Total		
	Point estimate	Lower bound	Upper bound	Point estimate	Lower bound	Upper bound	Point estimate	Lower bound	Upper bound
Amhara Region	199,929	122,889	321,230	443,975	296,386	654,405	643,904	419,274	975,635
Zones									
North Gondor	21,996	13,378	35,843	49,669	31,605	76,663	71,665	44,984	112,506
Waghemira	4,716	2,807	7,773	10,127	6,391	15,581	14,844	9,198	23,354
South Gondor	15,579	9,285	25,908	35,344	22,278	55,051	50,923	31,563	80,960
North Wollo	29,631	19,314	44,762	63,163	45,666	86,186	92,795	64,980	130,948
West Gojjam	42,729	23,540	74,338	88,644	56,656	133,936	131,373	80,196	208,273
Awi	9,531	6,337	14,274	21,699	15,286	30,557	31,230	21,622	44,832
East Gojjam	24,751	16,195	37,634	56,644	39,415	80,682	81,395	55,610	118,316
South Wollo	17,353	10,214	29,286	44,541	28,447	68,778	61,893	38,662	98,064
Oromiya	2,193	1,214	3,930	5,694	3,298	9,640	7,886	4,512	13,570
North Shewa	31,449	20,605	47,483	68,451	47,342	97,331	99,900	67,947	144,813

Lower and upper bounds represents the 95% confidence interval of the point estimates

## Discussion

This integrated survey of malaria and trachoma demonstrates that malaria is endemic in all zones of Amhara, an area of unstable malaria transmission, and that malaria parasites can be demonstrated at the end of the transmission season in a non-epidemic year. The key malaria indicators of mosquito net coverage and proportion of different population groups sleeping under long-lasting insecticidal nets (LLINs) demonstrated in the survey will be used as a baseline for planning and evaluation of the regional malaria control program. The survey has underscored the public health significance of blinding trachoma and allowed the calculation of intervention targets per zone with unprecedented precision. Conducting an integrated survey has maximized the benefits of organizing a complex survey without significant increases in the time required in the field or the effort of the field teams.

Selecting the sample and conducting field work posed considerable logistical challenges. The mountainous nature of Amhara makes transportation particularly difficult and field teams were obliged to walk for up to eight hours in order to reach some of the selected clusters. In accordance with the state definition of “malarious”, three urban centers (comprising 0.4% of the total population) were excluded from the sampling frame. By integrating malaria and trachoma, prevalence estimates, indicators, and risk factors for both diseases could be obtained for the cost of conducting one disease survey with an incremental addition of one person per field team. If the base survey were for malaria, the field team would require a trained trachoma examiner, if the base survey were for trachoma, the field team would require a clinical technician to take the blood slides. All interviewing, record-keeping, and form-checking would be constant.

In order to achieve the required sample size for the estimation of trichiasis, we required twice the number of people needed to estimate malaria parasite prevalence. We arbitrarily selected even-numbered households for malaria parasite prevalence and all households for trachoma. Since there were twenty-five households sampled and numbered sequentially per cluster, this resulted in systematically sampling 12 households for malaria parasite prevalence per cluster, or slightly less than half, and not half of all households. Such inadvertent compromises are likely to occur in integrated surveys and extremely careful planning is required to ensure that the overall objectives required to measure two or more outcomes are achieved. This integrated survey required considerably more pre-planning and coordination than a single disease survey, yet a relatively minor increase in implementation effort. The overall benefits of integration far outweighed the total effort required to plan, conduct and finance two separate surveys.

Amhara is prone to unstable epidemic malaria and the survey was conducted at the end of the transmission season in a non-epidemic year. The data presented here provide the first comprehensive representative baseline data of malaria parasite prevalence, species ratios and malaria indicators for the region. The parasite prevalence is consistent with that reported for unstable transmission areas in Ethiopia by Newman *et al* [21]. The Ethiopian Demographic and Household Survey conducted in 2005 estimated the proportion of households in Amhara that had any mosquito net to be 3.8% and the average number of LLINs per household to be 0.0 [22]. Our data suggest that by the end of 2006 considerable progress has been made in the Amhara Regional State malaria control program, as these indicators have risen to 34.7% of households with any net and a mean of 0.3 LLINs per household. In addition, these data show what needs to be accomplished if the national target of having everybody at risk of malaria sleeping under an insecticidal net, with a mean of two LLINs per household by the end of 2007 are to be achieved.

We included the use of rapid diagnostic tests (RDT) in the field component of the survey in order to maximize the likelihood that participants who were parasite positive received prompt and effective treatment. After the blood slides were read, it was possible to link back to the database and determine the proportion of participants positive by microscopy who were also positive by RDT. There was not absolute concordance between microscopy, which is considered the gold standard, and the RDTs. Participants with negative RDTs and positive blood slides were followed up back to the field and provided treatment according to national guidelines. The discordance will be presented in a separate paper.

Data collected in the survey are all indicative of the prevalence of the diseases and extent of indicators and risk factors. The prevalence of malaria is time-dependent and will vary from month to month and year to year. There is no known seasonality in presentation of clinical signs of trachoma in Ethiopia; however, the application of the simplified grading system is subjective. Clinical examiners went through a two-stage training and were required to have greater than 80% concordance with the gold standard before joining the field teams, which minimizes but does not exclude the risk of observer bias. It has been argued that nucleic acid amplification techniques of conjunctival swabs are the best way of determining the prevalence of ocular chlamydial infection, although this is currently advocated by the WHO for programmatic use [23].

The 2005/2006 National Survey on Blindness, Low Vision and Trachoma in Ethiopia had a total of 174 clusters of which 33 were in Amhara [2]. The sampling framework was based on probability proportional to size. A total of 4,609 individuals were surveyed but trichiasis was only reported for the adult population (persons aged 15 years and above) who comprise around 50% of the total population. The national survey estimated the TT prevalence in adults in Amhara to be 5.2%. Our survey included 160 clusters and a total of 17,242 persons surveyed for trichiasis. By having 10 domains and 16 clusters in each domain, rather than one domain and 33 clusters used in the national survey, we are able to estimate the trichiasis burden with greater precision, and have the possibility of focusing interventions to the zones of greatest need. Planning for interventions to operate the TT surgical backlog is conducted at the level of the zone. The estimates derived in this survey enable realistic preparation and planning. The threshold for district-wide intervention with mass antibiotic administration, hygiene promotion and environmental change is 10% TF in children aged 1–9 years [19]. The national survey estimated TF in children aged 1–9 years to be 39.1% in Amhara. Our survey found an overall weighted prevalence of 32.7%, ranging from 12.6% to 60.1% by zone, but more importantly for program planning, the threshold of 10% was exceeded in all ten zones.

The next steps in terms of integrating the delivery of the control program is to determine where integration makes sense in terms of logistics and to continue with separate activities where there are no potential synergistic gains from integration. For the distribution of LLINs, the FMOH policy of providing two free nets per household is not suitable for integration with the strategy for delivering azithromycin for trachoma control, since only one person per household needs to present themselves for nets whereas the entire household need to present themselves and be measured for mass drug administration. Specific training for health extension workers and village volunteers makes sense logistically and can be integrated. The program will use the findings from the malaria indicator questionnaire and trachoma risk factor questionnaire to design combined health education materials that include promotion of positive existing behaviours that will control malaria and trachoma.

The purpose of this paper is to present selected malaria indicators, malaria parasite prevalence, and key trachoma indicators to facilitate planning and evaluation of the Amhara Regional Health Bureau's integrated malaria and trachoma control program. Further analysis is underway to characterise the risk factors and identify the best opportunities for promotion of the integrated control program.

## References

[1] Federal Ministry of Health E (2005). Health and Health Related Indicators, [Ethiopian calender] 1997 ( = GC 2004–2005)..

[2] Berhane Y, Worku A, Bejiga A (2006). National survey on blindness, low vision and trachoma in Ethiopia..

[3] Burton MJ, Bowman RJ, Faal H, Aryee EA, Ikumapayi UN (2006). The long-term natural history of Trachomatous Trichiasis in The Gambia.. Invest Ophthalmol.

[4] Frick KD, Melia BM, Buhrmann RR, West SK (2001). Trichiasis and disability in a trachoma-endemic area of Tanzania.. Arch Ophthalmol.

[5] WHO, Deafness WHOPftPoBa (1997). Future Approaches to Trachoma Control..

[6] Abeku TA, van Oortmarssen GJ, Borsboom G, de Vlas SJ, JDF H (2003). Spatial and temporal variation of malaria epidemic risk in Ethiopia: factors involved and implications.. Acta Tropica.

[7] Cox J, Craig M, le Sueur DBS (1999). Mapping malaria risk in the highlands of Africa. MARA/HIMAL Technical Report..

[8] Negash K, Kebede A, Medhin A, Argaw D, Babaniyi O (2005). Malaria epidemics in the highlands of Ethiopia.. East Afr Med J.

[9] Federal Ministry of Health E (2006). Proceedings of the national workshop on review of the first five-year (2001–2005) and preparation of the second five-year (2006–2010) national strategic plan for malaria prevention and control in Ethiopia. Bekele Molla Hotel, Nazareth, 11–13 January 2006..

[10] Molyneux DH, Nantulya VM (2004). Linking disease control programmes in rural Africa: a pro-poor strategy to reach Abuja targets and millennium development goals.. Bmj.

[11] Hotez PJ, Molyneux DH, Fenwick A, Ottesen E, Ehrlich Sachs S (2006). Incorporating a rapid-impact package for neglected tropical diseases with programs for HIV/AIDS, tuberculosis, and malaria.. PLoS Med.

[12] Molyneux DH, Hotez PJ, Fenwick A (2005). “Rapid-impact interventions”: How a policy of integrated control for Africa's neglected tropical diseases could benefit the poor.. PLoS Medicine.

[13] Druilhe P, Tall A, Sokhna C (2005). Worms can worsen malaria: towards a new means to roll back malaria?. Trends Parasitol.

[14] BFEOD (2006). Population estimates by Ethiopia Bureau of Finance and Economic Development for the year 2006/2007. Addis Ababa..

[15] WHO (2005). Malaria Indicator Survey: Basic documentation for survey design and implementation. In: RBM/MERG, editor..

[16] Federal Ministry of Health E (2004). Malaria diagnosis and treatment: guidelines for health workers in Ethiopia (2nd Edition). Addis Ababa, Ethiopia..

[17] WHO (1991). Basic laboratory methods in medical parasitology..

[18] Thylefors B, Dawson CR, Jones BR, West SK, Taylor HR (1987). A simple system for the assessment of trachoma and its complications.. Bull World Health Organ.

[19] WHO (2006). Trachoma Control: A guide for program managers..

[20] STATA (2005). Stata survey data reference manual..

[21] Newman RD, Hailemariam A, Jimma D, Degifie A, Kebede D (2003). Burden of malaria during pregnancy in areas of stable and unstable transmission in Ethiopia during a nonepidemic year.. J Infect Dis.

[22] DHS (2006). Ethiopia demographic and health survey, 2005..

[23] Dawson CR, Schachter J (2002). Should trachoma be treated with antibiotics?. Lancet.

